# *In vitro* 3D microfluidic peritoneal metastatic colorectal cancer model for testing different oxaliplatin-based HIPEC regimens

**DOI:** 10.1515/pp-2023-0033

**Published:** 2024-02-28

**Authors:** Aras Emre Canda, Tolga Sever, Gizem Calibasi Kocal, Yasemin Basbinar, Hulya Ellidokuz

**Affiliations:** Institute of Oncology, Department of Translational Oncology, Dokuz Eylul University, Izmir, Türkiye; Institute of Oncology, Dokuz Eylul University, Izmir, Türkiye

**Keywords:** HIPEC, 3D microfluidic model, peritoneum metastatic colorectal cancer

## Abstract

**Objectives:**

Treatment of colorectal peritoneal metastases with cytoreductive surgery and hyperthermic intraperitoneal chemotherapy (HIPEC) is still evolving. Conducting a randomized trial is challenging due to the high heterogeneity in the presentation of peritoneal disease and various surgical approaches. Biological research may facilitate more rapid translation of information into clinical practice. There is an emerging need for a preclinical model to improve HIPEC treatment protocols in terms of drug doses and treatment durations. The aim of the study is to design a tool that serves as an *in vitro* three-dimensional (3D) microfluidic peritoneal metastatic colorectal cancer model to test the efficacy of different HIPEC treatments.

**Methods:**

We determined the effects of current therapy options using a 3D static disease model on human colon carcinoma cell lines (HCT 116) and transforming growth factor-β1 induced epithelial-to-mesenchymal transition (EMT) HCT 116 lines at 37 °C and 42 °C for 30, 60, and 120 min. We determined oxaliplatin’s half maximal inhibitory concentrations in a 3D static culture by using viability assay. Clinical practices of HIPEC were applied in the developed model.

**Results:**

EMT-induced HCT 116 cells were less sensitive to oxaliplatin treatment compared to non-induced cells. We observed increased cytotoxicity when increasing the temperature from 37 °C to 42 °C and extending the treatment duration from 30 to 120 min. We found that 200 mg/m^2^ oxaliplatin administered for 120 min is the most effective HIPEC treatment option within the framework of clinic applications.

**Conclusions:**

The tool map provide insights into creating more realistic pre-clinical tools that could be used for a patient-based drug screening.

## Introduction

Colorectal cancer (CRC) is the third most common malignancy and the second deadliest cancer. These cases and deaths account for approximately one in 10 of all cancer diagnoses and mortalities [1]. Metastasis is the primary factor leading to death in cancer patients, including those with CRC. The epithelial-mesenchymal transition (EMT) program endows metastatic properties such as invasion, mobility, and resistance to apoptotic stimuli [2]. CRC patients often have distant site metastases, including the lungs, liver, and peritoneal cavities. The peritoneal metastasis (PM) presents a worse prognosis compared to the other distant metastasis [3]. PM is challenging to diagnose with routine imaging techniques due to limited contrast resolution and small sizes [4]. Often, this form of metastasis obstructs systemic therapy and surgery, leaving palliative care as the only treatment option to maximize quality of life. Without palliative treatment, the median overall survival (OS) for these patients is approximately five months. Additionally, the effectiveness of systemic chemotherapy is reduced in CRC patients with PM (CRCPM), and poor visualization of the treatment outcomes limits the assessment of benefits. Therefore, intraperitoneal chemotherapy emerges as a promising local treatment option to target microscopic residual disease within the peritoneum [5].

Recently, CRCPM patients have undergone standard treatment involving Cytoreductive Surgery and Hyperthermic Intraperitoneal Chemotherapy (CRS & HIPEC). Although this dual treatment approach is determined by known prognostic factors, OS varies, and both mortality and morbidity are relatively high [6]. However, the recently published phase 3 PRODIGE 7 trial, which compared CRS plus oxaliplatin (Ox)-HIPEC with CRS alone, failed to show an overall survival benefit of adding HIPEC-Ox to CRS [7]. Another randomized trial, the COLOPEC trial, also failed to confirm the utility of prophylactic adjuvant HIPEC in patients with locally advanced colon cancer [8]. Clinical usage of Ox in HIPEC treatment is inconsistent; there are at least 12 different approaches to drug dosing and treatment duration [9].

A one-size-fits-all *in vitro* tool for various diseases or treatment applications is unfeasible. To acquire predictive preclinical data, a clear articulation of the biological question is necessary, along with identifying the model tool that aligns with the case-specific advantages and disadvantages [10].

Traditional 2D cultures have significant limitations, such as disrupted interactions between cellular and extracellular environments and changes in cell morphology, polarity, and division methods. These shortcomings have led to the development of models that more closely mimic *in vivo* conditions. On the other hand, patient-derived xenografts (PDX-orthotopic) are less suited for large-scale drug screening and personalized precision medicine due to their long turn-around time, high cost, and ethical limitations [11, 12]. There is growing evidence that *in vitro* three-dimensional (3D) tumor models with increased physiological relevance could enhance the predictive value of pre-clinical research and contribute to more timely decision-making in cancer therapy development. The inclusion of various elements of the tumor niche in cell model design is expected to yield more predictive *in vitro* tumor models [13]. Previous preclinical studies on Ox-HIPEC treatment *in vitro* CRCPM models are listed in Supplementary Table 1. In this study, we focus on non-cellular microenvironmental parameters like the extracellular matrix (ECM) and interstitial flow. The ECM comprises laminins, collagen IV, heparan sulfate proteoglycans, and various growth factors, cytokines, chemokines, and proteases [14]. Interstitial fluidic flow can influence cell behavior and response to chemotherapy. For this reason, we incorporated interstitial fluidic flow into our 3D model using a system previously published by our research group, Calibasi-Kocal et al. [15].

The study aims to develop a standardized model for assessing HIPEC treatment efficacy using a 3D microfluidic system that mimics CRCPM. This promising tool could assist in predicting the administration parameters for HIPEC treatment and potentially serve as a personalized drug screening method.

## Materials and methods

### Modelling metastatic colorectal cancer on microfluidic chip

**Figure 1:**
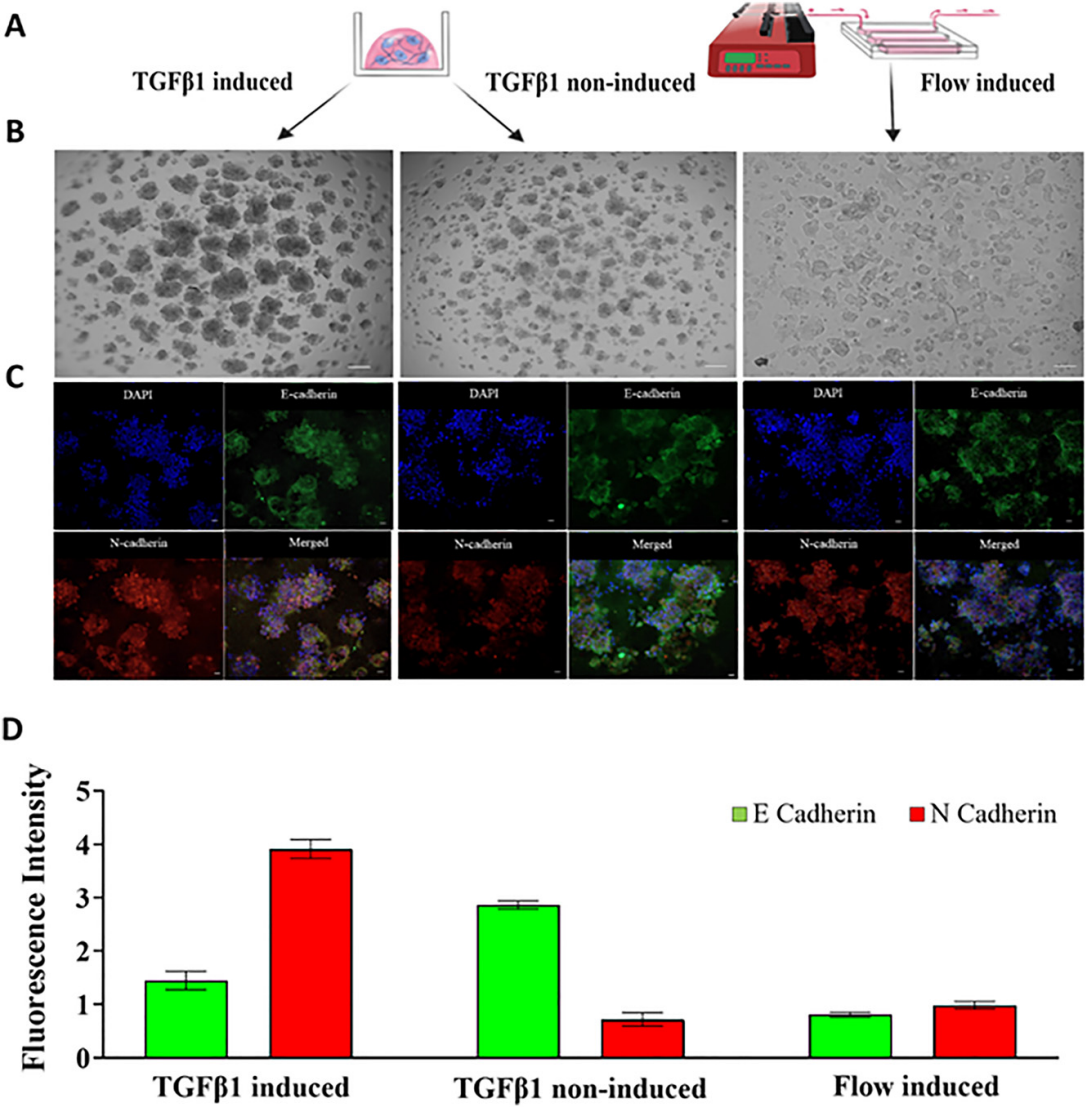
Method validation of the 3D CRCPM mimicking microfluidic model. (A) Static and microfluidic chip designs. (B) BF images of static and dynamic cultured cells (10×). (C) Immunofluorescent staining with DAPI and EMT markers E and N cadherin for TGFβ1 or flow rate induced EMT (DAPI: blue, E cadherin: green, N cadherin: red) (10×). (D) Fluorescent intensities of EMT markers. Treatments were triplicated (*p<0.05). Scale bar: 100 µm.

For this study, we employed modeling techniques for dynamic flow-based HIPEC treatment as well as microfluidic cell culture. We used a microfluidic chip previously developed by our group in the study by Calibasi-Kocal et al. designed to simulate an interstitial flow-based dynamic cancer microenvironment [15]. In this current study, this microfluidic chip-based cell culture system was modified in peritoneal metastatic lesions of CRC. Chip components, which included polymethyl methacrylate-PMMA (McMaster Carr, Santa Fe Springs, CA, USA) with thicknesses of 1.5 and 3.175 mm thickness, and 80 μm double-sided adhesive film (iTapeStore, Scotch Plains, NJ, USA), were assembled under aseptic conditions. Each chip features three channels (width: 4 mm, height: 27 mm) to culture CRC cells in a three-dimensional (3D) structure at a continuous flow rate of 2 μL/min. Human colorectal carcinoma cells HCT 116 (ATCC – American Type Culture Collection, CCL-247) were cultured in McCoy’s 5A (Biochrom, USA) media supplemented with 1 % fetal bovine serum (Biochrom, USA) and 1 % penicillin/streptomycin (Biochrom, USA) at 37 °C, 5 % CO_2_. Transforming growth factor-β1 (TGFβ1, R&D Systems, USA, 5 ng/mL) was applied for 48 h as an epithelial-mesenchymal transition (EMT) inducer to achieve metastatic phenotype of HCT 116 cells [16]. To assess the impact of EMT induction via TGFβ1 and flow rate, we performed E cadherin, and N cadherin immunofluorescent staining (Figure 1). Both HCT 116 and EMT-induced HCT 116 cells were trypsinized for 5 min using Trypsin/EDTA and embedded into a gelatinous basement membrane matrix (Matrigel Basement Membrane Matrix, Corning, USA). These cells were then mixed with MatriGel^®^ at different ratios – 1:10, 1:5, and 1:3 – to ascertain optimal robustness under flow conditions. We observed that a MatriGel^®^ ratio of 1:3 offered higher stability under flow, so all experiments were conducted using this ratio (Supplementary Figure 2). To attain a spheroid culture, 8×10^4^ cells in 1:10 cell suspension-to-Matrigel ratio was plated drop-wise at the base of the microfluidic chip channels (Supplementary Figure 1). The chips were then inverted and incubated at 37 °C in a humidified chamber until spheroid formation was visible. Finally, channels were covered with a 1:3 medium-to-Matrigel mixture, totaling 100 µL, gently added onto the droplets within the microfluidic channels. To solidify the Matrigel, the microfluidic chip was incubated for 30 min at 37 °C.

### HIPEC treatment on the CRC-chip model

After the gelation of Matrigel for 3D culture, silicon-based tubbing was connected to the inlet/outlet ports to simulate a flow-based dynamic peritoneal microenvironment and HIPEC treatment. Fresh medium was passed through the channels using a programmable syringe pump (NewEra Pump Systems Inc., USA) at a flow rate of 2 μL/min. The dynamic culture was maintained for 48 h prior to HIPEC treatment. Pre-determined IC_50_ doses of Ox at 37 °C (normothermia) and at 42 °C (hyperthermia) were administered for 30, 60, and 120 min to both TGFβ1 induced and non-induced HCT 116 colorectal cancer cell lines. Additionally, the 3D microfluidic culture model was tested with 460 mg/m^2^ for 30 and 60 min and 200 mg/m^2^ for 120 min to mimic the common clinical HIPEC protocols [17], [18], [19]. Dose conversions are detailed in Supplementary Method 1.3. All experiments were conducted in quadruplicate.

### Cell viability assay

To assess the cytotoxic effects of Ox, PrestoBlue^®^ Cell Viability Reagent (Thermo Fischer Scientific, USA) was applied at the 48th h. The assay was optimized for a 3D microfluidic culture system over 3 h (Supplementary Figure 2). After mimicking flow-based Ox HIPEC treatment, a viability assay was conducted. Fluorescence excitation (535 nm) and emission (615 nm) spectra were captured using a Varioskan Lux Reader (Thermo Fisher Scientific, USA) at the 48th h after the treatment. Fluorometric intensity data were transformed and analyzed using GraphPad Prism (GraphPad Holdings, USA).

### Immunofluorescence staining

Relative expression levels of E cadherin and N cadherin were assessed via immunofluorescence staining. Cells were fixed with 4 % paraformaldehyde (Electron Microscopy Sciences, Hatfield, PA, USA), and stained with mouse monoclonal antibodies against human E-cadherin and rabbit polyclonal antibodies against human N-cadherin (Abcam, USA). Cells were subsequently incubated with appropriate secondary antibodies: Alexa Fluor-488 goat anti-mouse and Alexa Fluor-568 goat anti-rabbit (Life Technologies, USA). Stained cells were examined under a fluorescence microscope (Zeiss, LSM 800 Confocal, USA). Fluorescence intensity was measured with Zen 2.1 software (Zeiss, Blue edition, USA), with n=4. Intensity data were transformed into graphs, and analyzed using GraphPad Prism 9 (GraphPad Holdings, USA).

### Ethical approval

The study received approval from the Non-Interventional Ethics Committee of Dokuz Eylul University, under approval decision number 2019/11-25.

### Statistical analysis

All experiments were performed in quadruplicates. Graphs in figures display data as the median, ranging from min to max. One-way ANOVA and Mann Whitney U, a non-parametric test, were used to determine the significance of differences between control and treated groups. Data analysis was conducted using GraphPad Prism software (GraphPad Holdings, USA). The threshold for statistical significance was set at p <0.05.

## Results

The objective of the current study was to develop an *in vitro* 3D CRCPM-mimicking microfluidic model to explore improvements in HIPEC therapy options. To establish this 3D microfluidic cell culture model, we tested Matrigel ratios of 1:10, 1:5, and 1:3 in quadruplicate. Our findings revealed that the 1:3 Matrigel-to-cell suspension ratio demonstrated greater robustness compared to other dilutions (Supplementary Figure S1A and B). The PrestoBlue cell viability assay was optimized for this ratio in a 3D microfluidic system across different incubation times – 0.5, 1.0, 1.5, 2.0, 3.0, and 5.0 h. An optimal incubation time of 3 h was identified (Supplementary Figure S1C and D).

**Figure 2:**
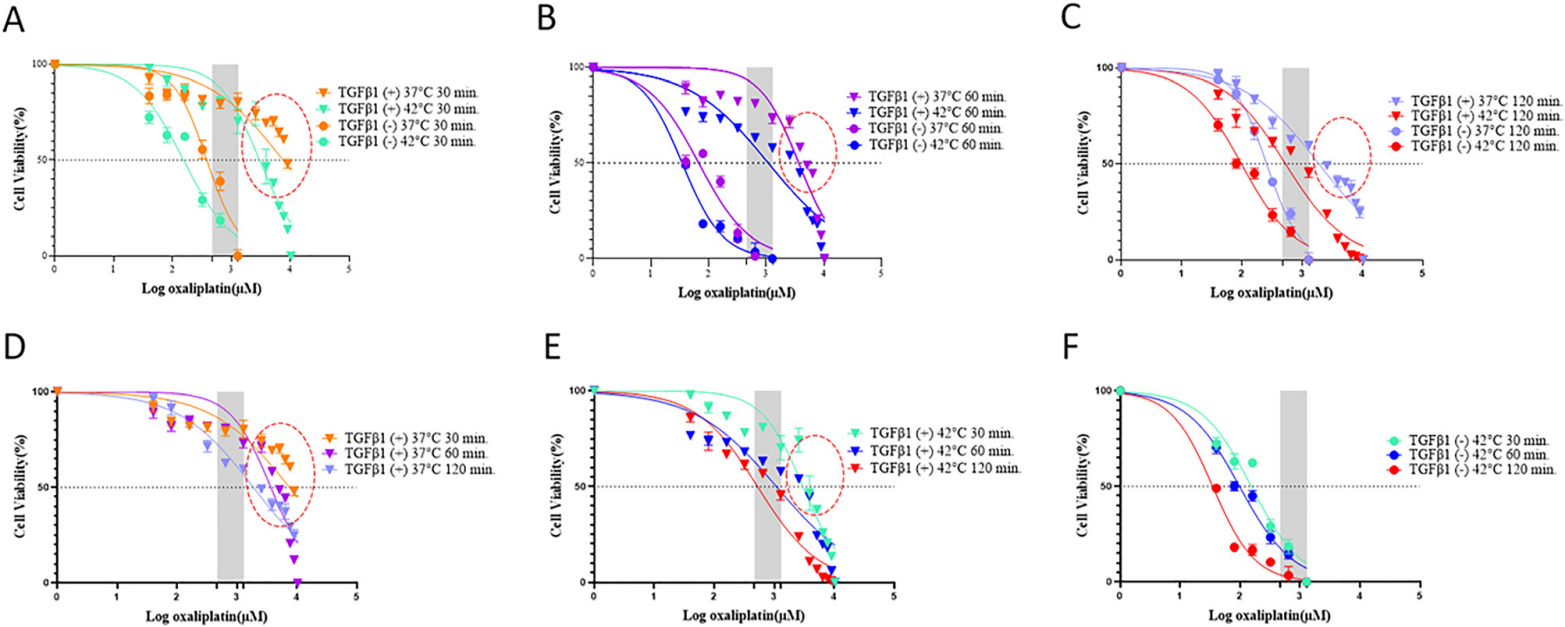
Dose–response curves for treatment groups. (A–C) Dose–response curves for 30, 60, and 120 min at 42 and 37 °C in TGFβ1 induced and non-induced HCT 116 cells. (D–F) Time-dependence efficacy at normothermic and hyperthermic conditions. Cell viability was assessed at 48 h post perfusion. Quadruplicated treatments. The shaded x-axis bar shows the Ox dose range (200–460 mg/m^2^).

We tested Ox IC_50_ doses for durations of 30, 60, and 120 min under both normothermic and hyperthermic on 3D static models involving TGFβ1 induced and non-induced HCT 116 cells. Dose–response curves for each treatment group are depicted in Figure 2. Figure 2A illustrates the temperature-dependent impact of a 30 min treatment. The data show that hyperthermia enhances Ox’s cytotoxic effect on both TGFβ1-induced and non-induced HCT 116 cells in the 3D static model. Figure 2B and C support this trend, indicating that hyperthermic conditions augment Ox-induced cytotoxicity in both cell groups. Based on the dose–response curves, IC_50_ doses of each treatment regimen were calculated (Supplementary Table S2). The table summarizes that TGFβ1-induced HCT 116 cells are approximately 10-fold less sensitive to Ox treatment in all scenarios except for 42 °C for 60 min, where sensitivity is induced 20-fold. Additionally, longer treatment durations the led to increased Ox-induced cytotoxicity, even at lower doses.

**Figure 3:**
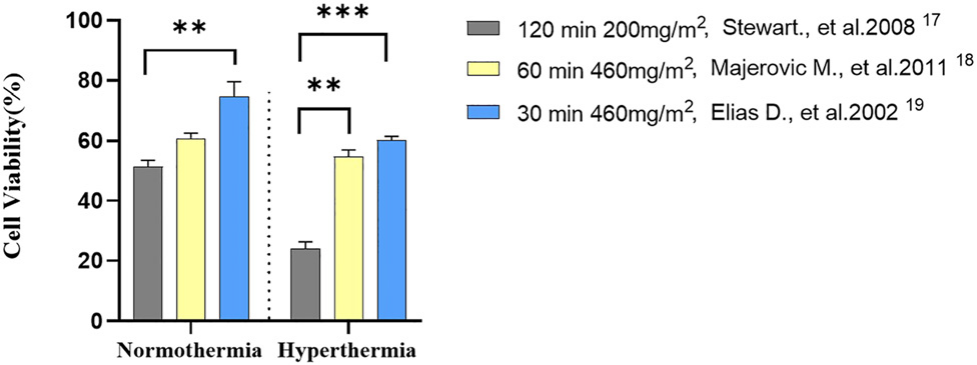
Evaluation of clinically relevant Ox-HIPEC treatment doses. Drug concentration converted to drug weight per square meter. Five replicates per treatment. Statistical significance was calculated using Mann Whitney U (*p<0.05, **p<0.01).

We also examined commonly administered clinical treatments for HIPEC, including those recommended by Elias et al. (460 mg/m^2^ for 30 min), Majerovic et al. (460 mg/m^2^ for 60 min), and Stewart et al. (200 mg/m^2^ for 120 min) using the 3D CRC-PM mimicking system [19], [20], [21]. Following these treatments, cell viability at the 48th h post-perfusion was 61.6 %, 54.6 %, and 24.9 %, respectively, under normothermic conditions. Under the same conditions, cell viability rates at the 48th h post-perfusion of 460 mg/m^2^ for 30 min, 460 mg/m^2^ for 60 min, and 200 mg/m^2^ for 120 min were 80.2 %, 69.7 %, and 49.3 %, respectively, as shown in Figure 3.

## Discussion

The established treatment for CRCPM patients combines CRS with HIPEC. CRS removes macroscopic tumors from the peritoneum and is supplemented by HIPEC to eliminate microscopic tumor residuals, enhancing treatment efficacy [20]. Despite this, HIPEC protocols vary in drug type, concentration, administration temperature, and duration [21]. Our goal is to develop a preclinical tool that mimics the CRCPM system and precisely predicts HIPEC administration parameters.

Conventional preclinical studies often rely on two-dimensional (2D) cell culture and animal models, each with its own set of limitations. Traditional 2D models fail to capture the complex microenvironment and structure of tumors, constraining their translational potential. More realistic *in vitro* models could not only improve pre-treatment evaluations but also reduce the need for animal testing, thereby adhering to the 3Rs (Replacement, Reduction, and Refinement) as recommended by Russel and Burch [22]. The 3D cell culture model incorporates cell–cell and cell–matrix interactions, offering a closer approximation to *in vivo* conditions. Additionally, microfluidic systems allow for the study of shear stress effects on cells. These advances make 3D microfluidic models more accurate for drug screening and could accelerate the pace of clinical studies [23, 24]. It is also worth noting that animals have physiological differences compared to human tumors. The shift towards 3D models aligns with the ethical imperative of minimizing harm to animals while still achieving robust scientific results. We’ve designed a 3D microfluidic model that mimics CRCPM to test and enhance the efficacy of HIPEC therapy, aiming to fill the gaps in current preclinical tools.

Numerous studies identified TGFβ1 as a key cytokine associated with the Epithelial-Mesenchymal Transition (EMT) program, which not only enables cancer cells to metastasize but also reduces their sensitivity to chemotherapeutic agents [25]. For example, Yin et al. found that TGFβ1-mediated EMT leads to reduced responsiveness to Ox in CRC [26]. In line with this, our study employed TGFβ1-induced HCT 116 cells as a model for CRCPM. We administered Ox under both normothermic and hyperthermic conditions to TGFβ1-induced and non-induced cells in a 3D static model for 30, 60, and 120 min. Our findings indicate that Ox’s-cytotoxic effects are more potent in TGFβ1 non-induced cells than in TGFβ1-induced cells. Upon determining the IC_50_ values of Ox for each treatment condition in the 3D static model, these doses were further evaluated in a 3D microfluidic model. Here, we observed a slight decrease in treatment efficacy in both TGFβ1-induced and non-induced groups, aligning with previous findings on the role of EMT in drug resistance in CRC by Mao et al. [16]. This consistency supports the necessity of dynamic culture systems for the development of preclinical tools that facilitate quicker translation of research to clinical practice. In our study, we considered TGFβ1 and interstitial fluidic flow as key factors that could alter cancer cell behavior in the tumor microenvironment, potentially affecting treatment outcomes. To focus on optimizing HIPEC treatment parameters like duration and dosage, we intentionally excluded other variable components, aiming for a less complex CRCPM disease model.

Research by Weinreich et al., underscores the importance of hyperthermia in boosting the cytotoxicity of Ox, attributing this to the inactivation of certain detoxification metabolism enzymes [27]. In line with this, our study found that TGFβ1-induced cells treated with Ox at normothermic conditions exhibited lower chemosensitivity compared to those treated under hyperthermic conditions. These observations echo findings by Atallah et al. who demonstrated that hyperthermia intensifies the cytotoxic effects of Ox in colorectal cancer cells [28]. Our findings are consistent with a report by Helderman et al. which stated that hyperthermia enhances the effectiveness of platinum-based drugs in a CRC static 3D organoid model. Furthermore, they posited that temperatures above 41 °C are necessary to significantly improve HIPEC efficacy [29]. To clarify, our data indicate that TGFβ1-induced cells treated at 42 °C for 30 min did not reach half-maximal inhibitory effects within the clinical dose range. On the contrary, cells treated for 60 and 120 min at hyperthermic conditions did achieve half-maximal inhibitory effects within the clinical dose range.

In our 3D CRCPM-mimicking microfluidic model, cells underwent HIPEC Ox treatments at various clinical dosages and durations (460 mg/m^2^ for 30 and 60 min, and 200 mg/m^2^ for 120 min). The data revealed that the 200 mg/m^2^ 120 min regimen resulted in the highest cytotoxicity, while the 460 mg/m^2^ 30 min regimen exhibited the lowest. The implications are noteworthy: lower dosage administrations translate to fewer side effects, a crucial consideration for clinical practice. Our findings align with a study by Forsythe et al. which showed that a 200 mg/m^2^, 120 min HIPEC Ox treatment enhances cytotoxic effects in CRCPM patient-derived tumor organoid static 3D cultures [30]. The PRODIGE 7 study further corroborated our observations, indicating that lowering the Ox dosage while extending the administration time to 120 min improves drug penetration through the peritoneal surface. It also emphasized the need to maximize hyperthermic conditions for optimal Ox cytotoxicity [7]. In a study by Kirstein et al., a 200 mg/m^2^, 120 min administration was more cytotoxic than a 460 mg/m^2^, 30 min regimen in a 2D cell culture system [31]. These diverse studies collectively validate our model as a reliable CRCPM simulation for investigating HIPEC treatments.

Two major limitations warrant mention and could serve as focal points for future research. The first limitation is the absence of certain microenvironmental cellular components, such as fibroblasts, vascular endothelial cells, and immune cells. These cell types significantly influence cancer cell survival, proliferation, and responsiveness to drugs [32]. The second limitation pertains to the culture system used. The study did not employ patient-derived tumor organoids, which are increasingly recognized for their versatility in drug screening and their ability to closely mimic original tumor tissue *in vitro* [33]. Utilizing such organoid models could provide more realistic preclinical tools and possibly refine current findings.

In summary, this research establishes the utility of a 3D microfluidic system for evaluating HIPEC treatment, particularly highlighting the synergistic effects of hyperthermia. The 3D CRCPM mimicking model presented here holds promise for integration with patient-derived organoid cultures in future investigations. This model offers the potential for predicting the most effective drug, dose, and treatment duration for CRCPM patients. Beyond its application in CRCPM, the microfluidic chip model is versatile enough for adaptation to different cancer types by simulating interstitial fluid flow. Correlating well with existing clinical data, this study serves as a stepping stone toward the standardization of methods for personalized, patient-derived therapies.

## Supplementary Material

Supplementary Material
