# Supplementary material for: In vitro 3D microfluidic peritoneal metastatic colorectal cancer model for testing different oxaliplatin-based HIPEC regimens
Source: Pleura Peritoneum. 2024 Feb 28;9(1):23–9. doi: 10.1515/pp-2023-0033 (PMC10980980; doi:10.1515/pp-2023-0033)
Supplement: Supplementary file 1 — Supplementary Material [file j_pp-2023-0033_suppl_001.docx]

**Supplementary**

**Supplementary Table 1.** Preclinical CRCPM models for Ox-HIPEC treatment.

- 1. **Spheroid Model Culture**

We optimized a protocol to culture HCT-116 cell spheroids in a microfluidic platform. Cells underwent 48-hour exposure to various Oxaliplatin concentrations at 37°C. Our work used a 3D microfluidic culture system (Figure 1). Spheroids were formed using a 1:10 matrigel-to-medium ratio and the hanging drop method on the base layer of the microchip. This took place in a humidified chamber over 2 hours. Each droplet housed 10 to 15 spheroids, each 150 to 250µm in diameter. Oxaliplatin drug screening indicated a dose-dependent effect on the spheroids, as shown in the related figure.


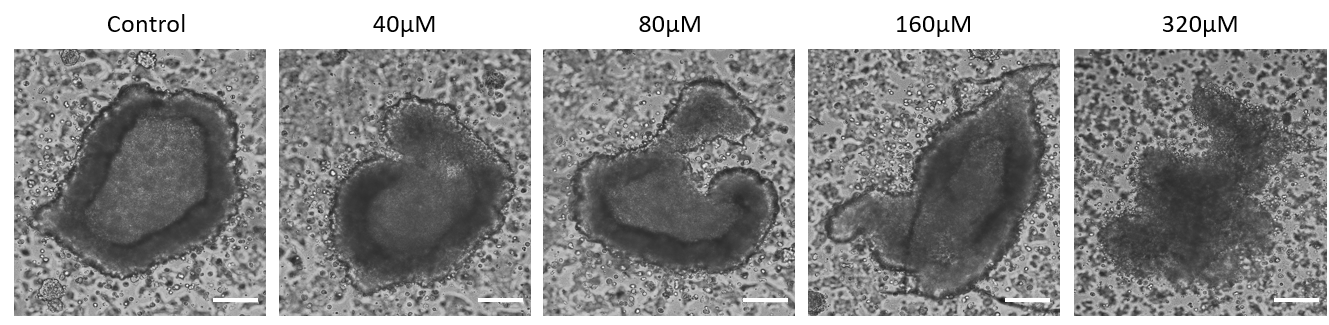


**Figure 1.** Bright-field micrograph shows spheroids situated in the microfluidic channels. An initial cell suspension of 8x10^4^ cells in a 1:10 Matrigel-to-medium ratio was pipetted onto the channel base. The system was inverted and incubated for 2 hours at 37°C. After the 1:3 Matrigel-to-medium mixture gelled, it was layered over the initial droplets. Subsequently, oxaliplatin concentrations (0, 40, 80, 160, and 320µM) in the culture medium were administered at 2µm/min using a precision pump for 48 hours. Final images were captured with an inverted microscope. Scale bar: 50µm.

- 1. **Characterization of 3D CRCPM mimicking microfluidic chip model**

A 3D microfluidic system was developed using varying ratios of Matrigel to colorectal cancer cells (8x10^4^ cells included) at 1:10, 1:5, and 1:3. The cells were spread on the bottom of the microfluidic system to assess their ability under glow conditions. Medium collected from the outlet was analyzed to determine the number of cells dislodged from the channels. To evaluate the integrity of Matrigel under these conditions, a Trypan Blue dye exclusion method was employed. Results related to Matrigel disruption are presented in Supplementary Figures 2A and B. Additionally, optimization of PrestoBlue cell viability reagent incubation time was carried out for the 3D microfluidic cell culture system. The maximum fluorescence intensity was observed at a 3-hour incubation period in a static 3D culture system, as shown in Supplementary Figure 2C. The design of the microfluidic chip is illustrated in Figure 2D. Fluid dynamics and shear stress characterizations are highlighted in Figures 2E and F.


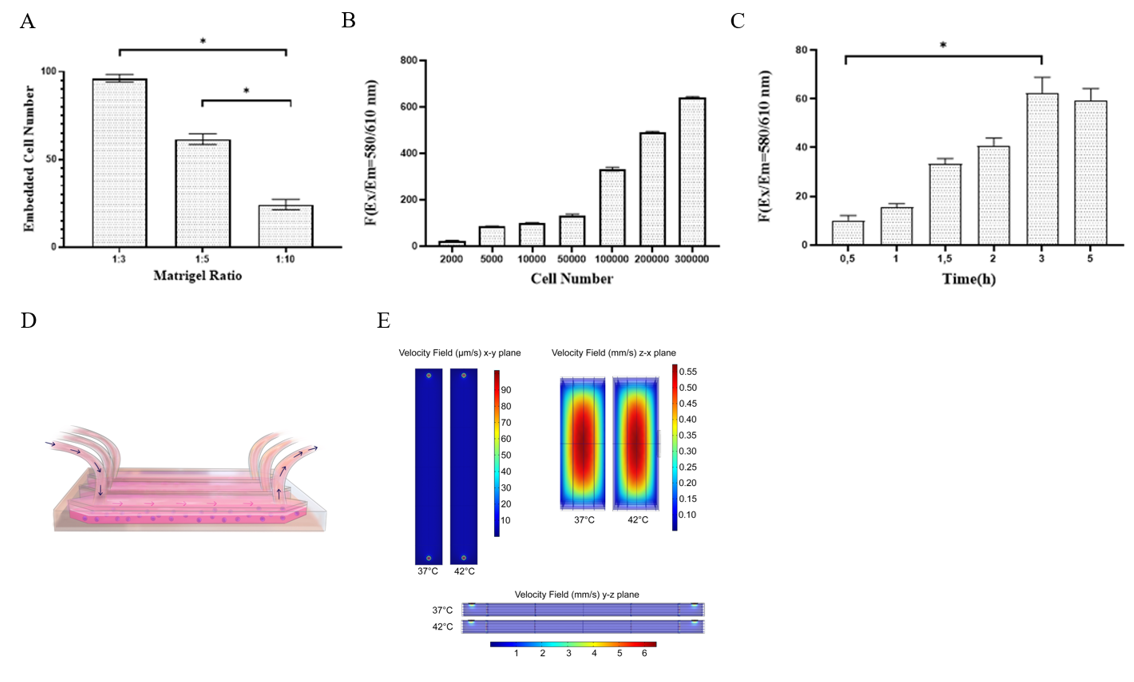


**Supplementary Figure 2.**

A. Examination of Matrigel stabilization in the 3D microfluidic model with varying ratios of 1:10, 1:5, and 1:3, each embedding 8x10^4 colorectal cancer cells, under flow conditions. B. PrestoBlue viability assay time course assessed on the 3D microfluidic model with differing cell densities of HCT 116 cells, ranging from 2x10^3^ to 3x10^5^. C. Determination of optimal incubation time for the PrestoBlue cell viability assay on a 3D microfluidic model incorporating 1x10^4^ HCT 116 cells, tested at time points ranging from 0.5 to 5.0 hours. All experiments were performed in quadruplicate. D. Detailed illustration of the compartments within the 3D microfluidic chip. E.  Computational Fluid Dynamics (CFD) simulations demonstrating the velocity field at the middle layer of a microchannel. Subsequent CFD simulations on the XY, YZ, and ZX planes confirm a uniform velocity field at the surface and sub-micrometer per second speeds at the bottom of the microchannel [11]. Statistical analysis was performed using the non-parametric Mann-Whitney U test, with *p < 0.05 deemed statistically significant.

- 1. **Efficacy of treatment dose in applied treatment conditions**

To ascertain the IC50 values of Oxaliplatin (Ox) under normothermic and hyperthermic conditions, a comprehensive range of doses was administered. For TGFβ1 non-induced HCT 116 cells, doses of 40, 80, 160, 320, 640, and 1280 µM were used. For TGFβ1 induced HCT 116 cells, an extended range of 40, 80, 160, 320, 640, 1280, 2560, 3840, 5120, 6400, 7680, 8960, and 10240 µM was applied. These doses were administered for durations of 30, 60, and 120 minutes in a 3D static culture system. All treatments were conducted in quadruplicate.

**Supplementary Table 2**. Efficiency of treatment dose for different treatments.

| **Treatment** | **IC50 Dose** | |
| --- | --- | --- |
|  | **TGFβ1 non-induced** | **TGFβ1 induced** |
| 37°C 30 min. | 972±13µM | 9374±151µM |
| 37°C 60 min. | 390±33µM | 5340±61µM |
| 37°C 120 min. | 338±17µM | 3225±96µM |
| 42°C 30 min. | 150±32µM | 2796±155µM |
| 42°C 60 min. | 96±32µM | 1246±45µM |
| 42°C 120 min. | 87±13µM | 645±32µM |

- 1. **Calculation of the clinical usage of HIPEC treatments**

Using an accepted large body surface area (BSA) of 1.8 m², drug concentration was converted to drug weight per square meter. This conversion was done to assess the impact of clinically relevant Ox-HIPEC treatments (460 mg/m² and 200 mg/m²) in the 3D CRCPM mimicking microfluidic model [11].

The channel's surface area was calculated as 1.08 cm², derived from channel dimensions of 0.4 cm in width and 2.7 cm in length. Post-Matrigel inoculation, the channel had a volume of 100 µl designated for the treatment solution.

Based on these calculations, a dose of 200 mg/m² corresponds to 500 µM, and 460 mg/m² equates to 1300 µM. These doses were prepared and applied to the CRCPM model to emulate the clinical application of HIPEC treatments.

**References**

1. Atallah D, Marsaud V, Radanyi C, Kornprobst M, Rouzier R, Elias D, et al. Thermal enhancement of Oxliplatin-induced inhibition of cell proliferation and cell cycle progression in human carcinoma cell lines. Int J Hyperthermia 2004;20(4):405-19.
2. Kirstein MN, Root SA, Moore MM, Wieman KM, Williams BW, Jacobson PA, et al. Exposure-response relationships for oxaliplatin-treated colon cancer cells. Anti-cancer drugs 2008;19(1), 37–44.
3. Ortega-Deballon P, Facy O, Jambet S, Magnin G, Cotte E, Beltramo JL, et al. Which method to deliver hyperthermic intraperitoneal chemotherapy with oxaliplatin? An experimental comparison of open and closed techniques. Annals of Surgical Oncology 2010;17(7), 1957–1963.
4. Piché N, Leblond FA, Sidéris L, Pichette V, Drolet P, Fortier LP, et al. Rationale for heating oxaliplatin for the intraperitoneal treatment of peritoneal carcinomatosis: a study of the effect of heat on intraperitoneal oxaliplatin using a murine model. Annals of Surgery 2011;254(1), 138–144.
5. Park EJ, Ahn J, Gwak SW, Park KS, Baik SH, Hwang SJ. Pharmacologic Properties of the Carrier Solutions for Hyperthermic Intraperitoneal Chemotherapy: Comparative Analyses Between Water and Lipid Carrier Solutions in the Rat Model. Ann Surgical Oncol 2018;25(11):3185–3192.
6. Ubink I, Bolhaqueiro ACF, Elias SG, Raats DAE, Constantinides A, Peters NA, et al. Organoids from colorectal peritoneal metastases as a platform for improving hyperthermic intraperitoneal chemotherapy. The British Journal of Surgery 2019;106(10):1404–1414.
7. Seyfried N, Yurttas C, Burkard M, Oswald B, Tolios A, Herster F, et al. Prolonged Exposure to Oxaliplatin during HIPEC Improves Effectiveness in a Preclinical Micrometastasis Model. Cancers (Basel) 2022;24;14(5):1158.
8. Forsythe SD, Sasikumar S, Moaven O, Sivakumar H, Shen P, Levine E, et al. Personalized Identification of Optimal HIPEC Perfusion Protocol in Patient-Derived Tumor Organoid Platform. Annals of Surgical Oncology 2020;27(13):4950–4960.
9. Liesenfeld LF, Hillebrecht HC, Klose J, Schmidt T, Schneider M. Impact of Perfusate Concentration on Hyperthermic Intraperitoneal Chemotherapy Efficacy and Toxicity in a Rodent Model. J Surg Res 2020;253:262-271.
10. Helderman RFCPA, Löke DR, Verhoeff J, Rodermond HM, van Bochove GGW, Boon M, et al.The Temperature-Dependent Effectiveness of Platinum-Based Drugs Mitomycin-C and 5-FU during Hyperthermic Intraperitoneal Chemotherapy (HIPEC) in Colorectal Cancer Cell Lines. Cells 2020;25;9(8):1775.
11. Calibasi Kocal G, Güven S, Foygel K, Goldman A, Chen P, Sengupta S, et al. Dynamic Microenvironment Induces Phenotypic Plasticity of Esophageal Cancer Cells Under Flow. Sci Rep 2016;6:38221.
